# Racial/Ethnic Patterns in Opioid Dispensing among Medicaid-Funded Young Children

**DOI:** 10.3390/healthcare11131910

**Published:** 2023-07-01

**Authors:** Laksika B. Sivaraj, Khoa Truong, William T. Basco

**Affiliations:** 1 Department of Public Health Sciences, Clemson University, Clemson, SC 29634, USA; lsivara@clemson.edu; 2 Addiction Medicine Center, Prisma Health, Greenville, SC 29601, USA; 3 Department of Pediatrics, Medical University of South Carolina, Charleston, SC 29425, USA; bascob@musc.edu

**Keywords:** prescription opioids, Medicaid, diseases of the respiratory system, injury, race/ethnicity

## Abstract

Racial differences in opioid dispensing for diseases of the respiratory system (RESP) and injury (INJURY) outpatient visits among patients ≤ 3 years old were examined. Outpatient claims data of South Carolina Medicaid children were analyzed over three three-year periods. The variable of interest was the triennial rate of dispensed opioid prescriptions per 1000 visits for RESP and INJURY diagnoses across racial/ethnic groups. Overall, dispensed opioid prescription rates related to RESP declined for all racial/ethnic categories. White children had the highest dispensing rate for RESP indications in the first period (5.6), followed by Black (4.5), and Hispanic (4.1). The likelihood of White children being prescribed opioids was higher than Blacks, and this was persistent over the studied time (rate ratios from 1.24 to 1.22, respectively). Overall opioid dispensing rates related to injury declined during the studied time. Hispanics had the highest dispensing rate for INJURY (20.1 to 14.8 to 16.1, respectively) followed by White (16.1 to 13.1 to 10.4, respectively). Relative differences in the dispensing rates across groups increased over time (Hispanics vs. White: rate ratios from 1.25 to 1.55, Hispanics vs. Black: from 1.52 to 2.24, and White vs. Black: from 1.24 to 1.44, respectively). There are considerable differences in the dispensing rates across racial/ethnic groups, especially in injury-related prescribing.

## 1. Introduction

Opioid prescribing among children accounts for only 2% of the overall prescription of opioids [1]. Yet, opioid exposure among children is considered high risk due to the increased toxicity of opioids with a lesser body mass, greater risk for side effects, and higher potential for misuse and abuse among adolescents and young adults. Findings from previous research suggest that pre- and post-natal opioid exposure can introduce long-term serious health risks, such as developmental delays, and psychological and mental health issues [2,3]. Opioid exposure in young children has been found to be associated with immediate and short-term adverse health outcomes, including, but not limited to, digestive system distress, respiratory depression, nervous system depression, poisoning, and even death [4,5,6,7,8]. The US Food and Drug Administration (FDA) and other professional societies discourage prescribing opioids to children [9,10,11]. Currently, the FDA requires revision of the safety labeling for prescription cough and cold medicines which contain codeine or hydrocodone in order to limit the use of these products to 18 years of age and older. Despite research findings and warnings from professional bodies, opioids continue to be prescribed to young children for non-indicated conditions [12,13,14,15].

While opioids are recommended to manage chronic pain or acute pain after a serious surgery or trauma [16,17,18], their prescribing for conditions not under those conditions is common among young children [12,13,14,15]. According to the 2017 South Carolina (SC) Medicaid data, 9% of the outpatient-related dispensed opioid prescriptions for young children (≤3 years) were for diseases of the respiratory system [12]. At the same time, 10% of the opioid prescriptions were written for injury-related acute pain management. As young children are prescribed considerable amounts of opioids for conditions that may not require opioids, such as respiratory illness, further understanding of the various aspects of opioid prescribing practices is needed to improve decision-making among both care providers and policymakers.

Past studies have indicated the presence of racial differences in prescribing opioids. These studies show that physicians in different settings are less likely to prescribe opioids to patients of a Hispanic ethnicity and/or Black and African American race [19,20,21,22,23,24,25]. The available literature on the racial/ethnic differences in pediatric opioid prescribing is mainly limited to emergency department settings, surgeries, or diagnoses associated with fractures [26,27,28,29,30,31,32,33]. Study findings associated with pediatric emergency department settings have suggested that physicians prescribe significantly fewer opioids but more non-opioids for non-Hispanic Black and Hispanic children compared to non-Hispanic White children [29,30,31]. Similar conclusions were made in these studies conducted among children with fractures [26,27,32,33]. It is possible that these differences in opioid prescribing are not solely based on the patient’s race/ethnicity but are instead due to factors associated with race/ethnicity, such as differences in cultural views and communication barriers [34].

We completed a previous study that revealed more than 35% of outpatient opioid dispensing among <3 years old patients in SC were related to diseases of the respiratory system (RESP) [12], even though opioids prescribed for RESP have long been considered contraindicated [15]. While opioids for injuries are indicated but discouraged for young children, we found that injury-related (INJURY) opioid dispensing accounted for around 8% of opioids dispensed to the same cohort [9,10,11]. In this study, we sought to further the understanding of opioid prescribing to young children by comparing opioid prescribing for two groups of diagnoses respiratory and injury seeking to evaluate the potential racial and ethnic differences in prescribing for conditions that should not be associated with opioids (respiratory), and conditions for which opioids are potentially more appropriate (injury). Given the high risks of opioid exposure among younger ages, our study focused on a very young age group, 1–36 months old. We have assessed two major research questions in this study:

1. Whether the rates of opioid dispensing are different across race/ethnicity groups at any point in time, for each of the two diagnoses studied;
2. Whether the gaps in opioid prescribing changed over time among race/ethnicity groups, again for each of the two diagnoses studied.

## 2. Materials and Methods

### 2.1. Study Population

We conducted a retrospective cohort study utilizing SC Medicaid claims data from the year 2009 to 2017, respectively. We used 2 main datasets: a paid-claims pharmacy dataset, which included information regarding the drug dispenses of Medicaid enrollees; and a paid-claims visit dataset, which included information regarding the outpatient visits of these enrollees along with the primary billing diagnoses related to each visit. These are restricted data, meaning they are not available to the public. Access to the data was approved by the South Carolina Data Oversight Review Council on a case-by-case basis. The proposed study was deemed as ‘not human research’ by the Institutional Review Board at the Medical University of South Carolina. Therefore, informed consent from the Medicaid-funded children was not required.

Children aged 1–36 months at the time of their outpatient visit were included. Children less than 1 month old were excluded due to the potential of infants being treated with opioids for neonatal opioid withdrawal syndrome [3].

### 2.2. Case Identification

Opioid dispenses were identified from the pharmacy dataset using the drug codes and drug names available in the pharmacy dataset. The National Drug Codes (NDC) of all drug products dispensed 2009–2017 (based on the Centers for Disease Control and Prevention (CDC) Opioid Overdose Indicator Support Toolkit and Red Book sources) were used to identify the patients with opioids prescribed [35,36]. We also utilized published lists of opioids used in other studies along with a search of the “drug name” variable for nomenclature conventions that typically identify opioids (e.g., “HC” for “hydrocodone”) [37]. We only focused on oral opioid preparations, meaning therefore that any injectable, patch, and ophthalmic preparation opioids were excluded from the analysis.

Once these dispenses were identified in the pharmacy dataset, they were linked with the visit dataset to determine the indication associated with opioid dispensing. Here, each outpatient visit was linked to any opioid prescription dispensed within the next 7 days after the visit date, following an approach utilized in the literature [38,39]. Linking an ambulatory visit to an opioid dispensing event was performed using the unique pseudo patient identifier provided in each dataset, along with the time variables available from both the pharmacy and visit datasets. Any opioid dispensing event that failed to form a link based on this 7-day criteria was subsequently excluded from the study.

We limited cases to outpatient visits only. The possible outpatient visits were identified based on the Healthcare Common Procedure Coding System (HCPCS codes) and limiting the filing type of the visit claims data to either be high-care (HIC) or outpatient (OP). These outpatient visits were further limited to ambulatory visits, emergency department visits, urgent care visits, and ambulatory surgery and procedure visits. Then, the primary diagnoses linked to these outpatient visits were categorized using the available International Classification of Diseases (ICD), 9th and 10th Revision primary diagnosis codes, as well as the multi-level Agency for Healthcare Research and Quality developed Clinical Classifications Software (CCS) [40].

**Figure 1:**
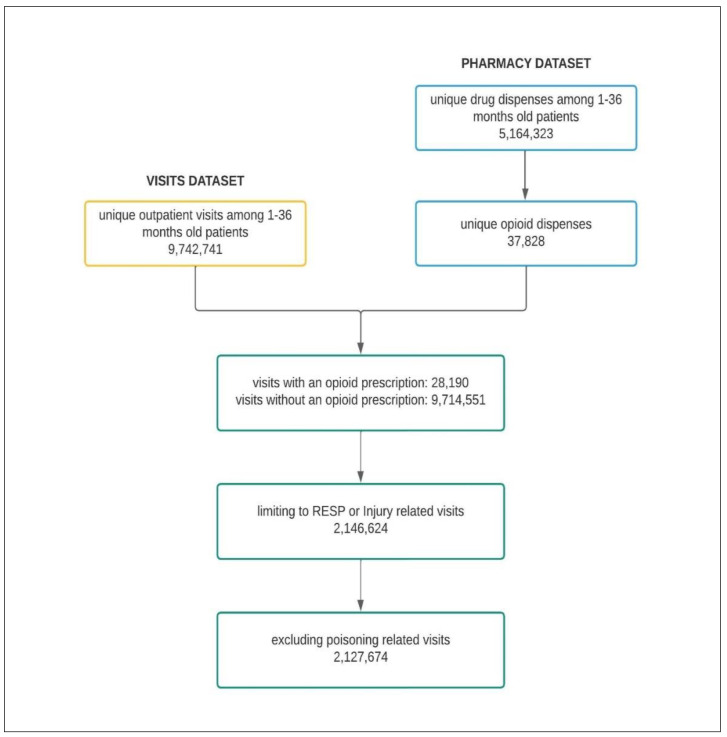
Inclusion and exclusion chart for identifying outpatient visits between the years 2009–2017 of 1–36 month old patients with respiratory system or injury diagnoses and related opioid dispensing.

The identified multi-level CCS categories were used to isolate the outpatient visits related to “INJURY” and “RESP”. The diagnoses related to “poisoning” were excluded from the “injury” category [41]. A detailed inclusion and exclusion chart has been presented in Figure 1. The patient’s race/ethnicity was categorized. SC Medicaid data recorded race and ethnicity in a combined format, and this data included 10 race/ethnicity groups; (1) “White and Caucasian”, (2) “Black or African American”, (3) “More than one race”, (4) “Federally Recognized Native American”, (5) “Other Native American”, (6) “Alaska Native”, (7) “Asian”, (8) “Other/Unknown”, (9) “Native Hawaiian/Pacific Islander”, and (10) “Hispanic”, respectively (Table 1). For analysis purposes, children with “Federally Recognized Native American”, “Other Native American”, “Alaska Native”, “Asian”, “More than one race”, and “Other/Unknown” were grouped together as “Other”. Children with missing race/ethnicity were included in our analysis as the “Missing” race/ethnicity group. After this regrouping, there were 5 race/ethnicity groups, which were as follows: (1) “Non-Hispanic White and Caucasian” (White), (2) “Non-Hispanic Black or African American” (Black), (3) “Hispanic”, (4) “Other”, and (5) “Missing” (Table 2), respectively. Racial category data were either self-reported by enrollees or their proxies at the time of enrollment, and enrollees were allowed to choose not to provide their racial or ethnic data.

### 2.3. Statistical Analysis

The outcome of interest was the triennial rates of dispensed opioid prescriptions per 1000 visits for each racial group within the RESP and INJURY diagnostic categories separately, using Equation (1).

Dispensed opioid rate per 1000 race/ethnic group-related visits for a diagnostic category per 3-year period =
(Number of opioid prescriptions linked to the race/ethnic group for a diagnostic category per 3-year period) × 1000/
(Number of visits related to the race/ethnic group for a diagnostic category per 3-year period)(1)

Once the outcome variable was calculated for each race/ethnic group for every 3 years, they were then compared across consecutive 3-year periods: 2009–2011 (first period), 2012–2014 (second period), and 2015–2017 (third period), respectively. This was performed separately for both RESP- and INJURY-related outpatient visits. Comparisons were made using proportional tests to identify significant changes at a 95% confidence level.

## 3. Results

After the application of the inclusion and exclusion criteria (Figure 1) the final dataset consisted of 2,127,674 RESP- or INJURY-related outpatient visits between the years of 2009 and 2017 of children 1–36 months old (Table 2). According to the data over the nine-year period, children had fewer outpatient visits related to the INJURY (295,402; 13.88%) when compared with the RESP (1,832,272; 86.12%), but INJURY visits demonstrated a higher overall (i.e., for the entire years included in the study) opioid dispensing rate (INJURY: 11.19 per 1000 visits and RESP: 3.14 per 1000 visits, respectively). There was a total of 9059 opioid dispenses linked to these INJURY- or RESP-related visits, and out of these opioid dispenses, 63.5% were linked to RESP-related visits, and the remainder were linked to INJURY-related visits. Most of these outpatient visits were from White patients (32.70%) and Black patients (30.79%). Only 7.48% of the visits were made by Hispanic patients, while 11.35% were from other race groups. As shown in Table 2, a high proportion of outpatient visits (17.68%) did not have information on the race of the patients (and thus a race-missing group was created instead of being excluded from our analyses).

### 3.1. Opioid Dispensing Patterns in the Diseases of the Respiratory System

**Figure 2:**
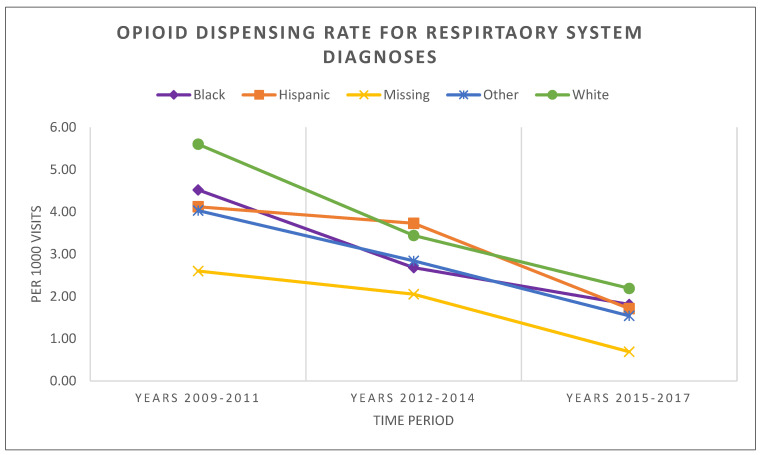
Patterns in opioid dispensing across racial/ethnic groups among South Carolina Medicaid outpatient visits from 1–36 month old patients related to respiratory system (RESP) diagnoses.

As shown in Figure 2 and Table 3, the overall dispensed opioid prescription rates related to RESP declined significantly for all racial/ethnic categories during the studied period. At any point in time, dispensing rates for RESP differed across these racial/ethnic groups with the gaps generally converging over time. Compared to the rest of the race/ethnic groups, the Hispanic and Missing groups had considerably lower rates until the period 2012–2014. White children had the significantly highest RESP opioid dispensing rate in the first 3-year period with 5.6 per 1000 visits, which significantly decreased to 2.2 per 1000 visits in the last 3-year period (*p*-value < 0.001), respectively. This was followed by Black (from 4.5 to 1.8), and Hispanic children (from 4.1 to 1.7). The likelihood of White children being prescribed opioids was significantly greater than that of Black children during the first and second periods. Even though this difference was not determined to be significant during the third period, the difference continued to persist over the study time. Within the first 3-year period the rate ratio was 1.24 (*p*-value < 0.001), while at the end of the period, it was 1.22 (*p*-value = 0.059). Even though White children had a lower opioid dispensing rate compared to Hispanic children during the second period, the difference observed between these groups became less significant by the end of the study period. The rate ratio within the first period was 1.36 (*p*-value < 0.001), and by the third period, it decreased to 1.28, indicating no significant difference between these rates. Furthermore, RESP-related opioid dispensing continued to decrease among the children whose race/ethnicity data was not collected. Even though the rate ratios of these children compared to White, Black, and Hispanic children declined over time, these children with their missing race/ethnicity continued to receive significantly fewer opioids. The results of the statistical testing of these triennial rates are listed in Appendix A Table A1 and Table A2, respectively.

### 3.2. Opioid Dispensing Patterns in Injury Conditions

**Figure 3:**
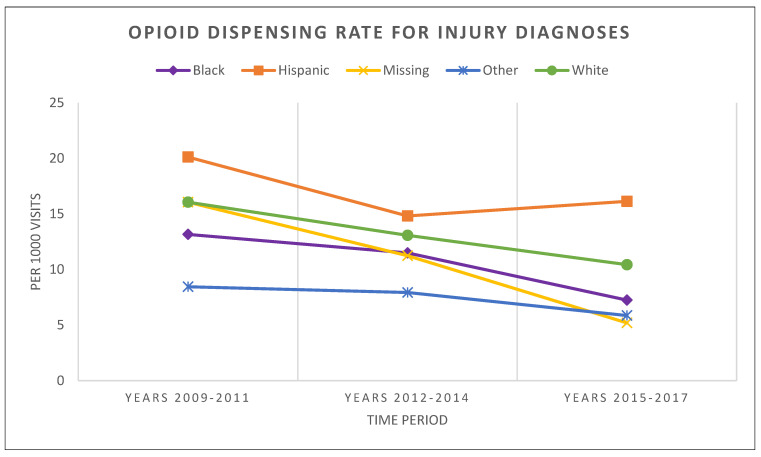
Patterns in opioid dispensing across race/ethnic groups among South Carolina Medicaid outpatient visits from 1–36 months old patients related to injury diagnoses.

The overall dispensed opioid prescription rates related to INJURY declined for all the race/ethnic groups assessed during the studied years (Figure 3 and Table 3). Similar to the trends in respiratory conditions at any point in time, dispensing rates differed across race/ethnic groups and the gaps between these groups generally persisted over time. Hispanic children had the highest opioid dispensing rate related to INJURY visits. This rate changed from 20.1 per 1000 injury visits (first period) to 14.8 (second period, with a *p*-value of 0.017) to 16.1 (third period), respectively. The second-highest opioid dispensing rate related to INJURY visits was for White children. This rate declined significantly from 16.1 to 13.1 (*p*-value < 0.001) to 10.4 (*p*-value = 0.004) per 1000 injury visits, respectively. However, the relative differences in the opioid dispensing rates across the main race/ethnicity groups increased over time. As Hispanic children were significantly more likely to receive opioids for INJURY compared to White and Black children at all time periods, the rate ratios also increased over time. Compared between Hispanic and White children, the rate ratio at the beginning was 1.25 (*p*-value = 0.012), which later increased to 1.55 (*p*-value = 0.008). When comparing Hispanic to Black children, the rate ratio at the beginning was 1.52 (*p*-value < 0.001), which then increased to 2.24 (*p*-value < 0.001). And when comparing White children to Black, White children were more likely to receive opioids during all three time periods (rate ratio of 1.24 with a *p*-value of < 0.001 at the beginning vs. 1.44 with a *p*-value of 0.001 at the end, respectively). Furthermore, INJURY-related opioid dispensing continued to decrease among the children whose race/ethnicity data was not collected, but their likelihood of receiving opioids compared to White, Black, and Hispanic children declined at an increasing rate. The results of the statistical testing of these triennial rates are listed in Appendix A Table A1 and Table A3, respectively.

## 4. Discussion

Evaluating opioid prescribing in very young children is crucial due to the relatively greater risk of adverse events compared to older children. Younger ages are inversely associated with the risk of opioid-related adverse events after opioid exposure, especially due to the respiratory and neurological suppression effects of these drugs. For that reason, the American Academy of Pediatrics has strongly discouraged opioid prescribing for children, and successive FDA rulings since the 2000s have sought to limit opioid prescribing to children under 18 for all conditions, all of which have likely contributed to the general downward trends observed in opioid prescribing and dispensing for the conditions studied [9,12,13,14,15,42].

Nationally, rates of opioid prescribing/dispensing to children have been declining as have been found in studies evaluating national trends and state-specific data [12,13,42,43,44,45]. Our evaluation of opioid prescribing for diseases of the respiratory system and injury conditions similarly demonstrated notable declines from the years 2009 to 2017, respectively. While it is reassuring to see overall declining trends, one would hope that the rates are declining even more for conditions where opioid prescribing is not indicated (in our case, respiratory conditions) compared to other conditions such as post-injury, for which opioid prescribing may be appropriate. With this study, we found reductions in opioid dispensing for both conditions—conditions in the respiratory system and injuries, but there were persistent gaps observed in the dispensing rates across the racial and ethnic groups assessed even as the overall opioid dispensing for these two groups of diagnoses was declining.

Overall, our study demonstrates that racial/ethnic differences in opioid dispensing related to RESP diagnoses are in line with the wider pediatric opioid prescribing and dispensing literature. For example, other studies have demonstrated that White children are more likely to receive opioids overall after any visit to emergency departments, after injury fractures, and after common pediatric surgeries [26,27,28,29,30,31,32,33]. One major finding from our study that differs from the literature is that the young Hispanic children in the current study were more likely than children of other racial or ethnic groups to receive opioids after an injury-related visit. One study indicated that among children <20 years old with long-bone fractures, non-Hispanic White children were significantly more likely to receive opioids compared to other race/ethnic groups [32]. Further, among those less than 18 years old with emergency department visits related to limb fractures, non-Hispanic White children were again more likely to receive opioid analgesia compared to non-Hispanic Black children, and Hispanic children were less likely [33]. One of our previous studies which was conducted using 20 years of SC Medicaid data demonstrated that among children less than 19 years old with limb fractures, Hispanic children were less likely to receive opioids compared to non-Hispanic White children [26], suggesting that the higher rates we found among young Hispanic children may represent an anomalous association based on the limited age range we studied and/or related to the two conditions assessed in this younger age group. Evaluation of our same research questions among all SC Medicaid children could have been more consistent with the other studies that demonstrated lower opioid prescribing to Hispanic children [26,27,29,30,31,32,33]. Furthermore, based on our study findings, it was evident that children with no reported race/ethnicity are less likely to receive opioids compared to White, Black, and Hispanic children. Given that parents and children may voluntarily decline to provide race or ethnic data to SC Medicaid, we feel that it is important to keep this group as a separate group in analyses and not just exclude them [26].

The reasons for these observed differences in opioid dispensing by race and ethnicity are likely multifactorial. Implicit bias certainly plays a role, as multiple studies have demonstrated that clinicians are less likely to prescribe opioids to minority patients [22,23,24,25]. However, patient belief factors may also play a role, and several studies have shown that Hispanic patients and their families may be less accepting of opioids overall. Ironically, the disparity in RESP dispensing that we have documented in this study may actually be beneficial to minority children, given that dispensing for respiratory system-related diagnoses occurs so often, and generally should be avoided. By evaluating only Medicaid-funded children, we expected to reduce the bias associated with socio-economic status (SES). It is not possible in claims data to know the degree to which the cough symptoms in each respiratory illness visit were severe enough for a provider to consider prescribing opioids, but the fact that minority children were less likely to receive opioids for any respiratory visit may actually represent a benefit. It is, however, more concerning to see the disparity among injury visits, the group of visits where pain mitigation, including potential opioids, maybe most indicated. In addition to documenting that the disparities in opioid dispensing for INJURY existed, we also documented that the relative rates negligibly changed among the racial and ethnic groups assessed.

Limitations of this study include the fact that these data come from one state and are limited to Medicaid-funded children within that state. However, the findings are largely consistent with findings from other studies in regard to documenting persistent racial disparities in opioid prescribing and dispensing among children. Even though we followed the pre-established linking criteria for the Medicaid datasets, due to the deidentification of the data, pharmacy claims data cannot be directly linked to the corresponding hospital visit or the diagnosis. And our limited data set does not include the patient, provider, or pharmacy location as a means of further reducing the risk of individual enrollee identification. This lack of geographic information did not allow for further exploration of the regional or health-system related differences that might have partially explained the differences between minority children vs. non-Hispanic White children. Race and ethnicity are self-reported in the South Carolina Medicaid data, but we do not, however, have the ability to determine the percentage of the racial and ethnic selections that were entered by a parent or patient versus being entered by an enrollment specialist with parental or patient input. Our previous study has indicated that the racial and ethnic categorization of a subject within the South Carolina Medicaid data is remarkably stable over time [26] nonetheless. Furthermore, pharmacy claims data only includes the dispensing information on the medication. And these dispensed amounts can be different from the prescribed amount, or the amount administered to the patients, information which we do not have the access to. Finally, there can be large regional variations in opioid prescribing and dispensing as has been clearly demonstrated among adult populations, but also seen in child populations [46,47,48], suggesting that one must be careful when trying to generalize our findings to Medicaid-funded children in other states or regions of the United States.

## 5. Conclusions

Among South Carolina Medicaid-funded children aged 1–36 months old, opioid dispensing for respiratory and injury diagnoses declined between the years 2009 and 2017, respectively. However, notable and persistent racial and ethnic differences in opioid dispensing were identified despite the overall decline in opioid dispensing. Racial and ethnic differences in opioid dispensing were observed when evaluating visits associated with injury diagnoses and for respiratory diagnoses, even though opioids for injuries may be considered appropriate while opioid prescribing for respiratory illnesses in young children is strongly discouraged. Time alone has not reduced these racial and ethnic opioid dispensing disparities among these young children, and further research that can better evaluate the contextual reasons for these differences will be needed.

## Figures and Tables

**Table 1:** Racial/ethnic groups of South Carolina Medicaid outpatient visits from 1–36 month old patients related to injury or respiratory system diagnoses during the years of 2009–2017.

Race/Ethnicity Groups ^a^	Number of Outpatient Visits (%)
White and Caucasian	695,750 (32.70)
Black and African American	655,025 (30.79)
Hispanic	159,105 (7.48)
Asian	7471 (0.35)
Federally Recognized Native American	965 (0.05)
Native Hawaiian or Pacific	404 (0.02)
Other Native American	4163 (0.20)
Alaska	21 (0.00)
Having more than one race	14,264 (0.67)
Other/unknown	214,287 (10.07)
Missing	376,219 (17.68)

^a^ South Carolina Medicaid data does not separately attribute ethnicity and race, and therefore “Hispanic” ethnicity has been utilized as a “racial” category.

**Table 2:** Characteristics of South Carolina Medicaid outpatient visits from 1–36 month old patients related to diseases of the respiratory system (RESP) or INJURY diagnoses during the years of 2009–2017.

Baseline Characteristic	Total (n = 2,127,674)	RESP (n = 1,832,272)	INJURY (n = 295,402)
Opioid-related	9059 (0.43)	5752 (0.31)	3307 (1.12)
Age (months) at outpatient visit: mean (SD)	16.95 (10.18)	16.47 (10.18)	19.94 (9.69)
Gender: n (%)			
Male	1,167,807 (54.89)	1,006,796 (54.95)	161,011 (54.51)
Female	959,834 (45.11)	825,448 (45.05)	134,386 (45.49)
Race/Ethnicity: n (%)			
Non-Hispanic White and Caucasian	695,750 (32.70)	591,129 (32.26)	104,621 (35.42)
Non-Hispanic Black and African American	655,025 (30.79)	564,227 (30.79)	90,798 (30.74)
Hispanic	159,105 (7.48)	142,184 (7.76)	16,921 (5.73)
Other ^a^	241,575 (11.35)	204,741 (11.17)	36,834 (12.47)
Missing	376,219 (17.68)	329,991 (18.01)	46,228 (15.65)

^a^ The updated “Other” race/ethnic category includes outpatient visits related to “Asian”, “Alaska”, “Federally Recognized Native American”, “Other Native American”, “Native Hawaiian or Pacific”, “More Than One Category”, and “Other/Unknown” race/ethnic categories from the original Medicaid SC data.

**Table 3:** Rate per 1000 visits for respiratory system and injury diagnoses during the years of 2009–2017.

Diagnostic Category		RESP			INJURY		
Time Period		2009–2011	2012–2014	2015–2017	2009–2011	2012–2014	2015–2017
Race/ethnic group	White	5.60	3.44 *	2.19 *	16.06	13.08 *	10.44 *
	Black	4.52 ^a^	2.68 ^a,^*	1.81 *	13.16 ^a^	11.49 ^a,^*	7.25 ^a,^*
	Hispanic	4.12 ^a^	3.73 ^b^	1.71 *	20.12 ^a,b^	14.82 ^b,^*	16.13 ^a,b^
	Other	4.03 ^a^	2.84 ^a,c,^*	1.54 ^a,^*	8.45 ^a,b,c^	7.94 ^a,b,c^	5.86 ^a,c^
	Missing	2.60	2.05 ^a,c^	0.69 ^a,b,c,^*	16.06	11.24	5.20 ^a,b,c,^*

^a^ Denotes a significant change in the rate per 1000 visits in the race/ethnic groups “Black”, “Hispanic“, “Other“, and “Missing“ compared to “White“ in each time period at a 95% confidence level. ^b^ Denotes a significant change in the rate per 1000 visits in the race/ethnic groups “Hispanic“, “Other“, and “Missing“ compared to “Black“ in each time period at a 95% confidence level. ^c^ Denotes a significant change in the rate per 1000 visits in the race/ethnic groups “Other“ and “Missing“ compared to “Hispanic“ in each time period at a 95% confidence level. * Denotes a significant change in the rate per 1000 visits in a given time period compared to the first period for a given race/ethnic group at a 95% confidence level.

## Data Availability

Not applicable.

## Appendix A

**Table A1:** A *p*-value table for the opioid prescribing rate difference in each time period compared to immediately before time period by racial/ethnic groups and diagnosis categories.

Race/Ethnic Group	RESP		INJURY	
	Second Period vs. First	Third Period vs. Second	Second Period vs. First	Third Period vs. Second
White	**<0.001**	**<0.001**	**<0.001**	**0.004**
Black	**<0.001**	**<0.001**	**0.040**	**<0.001**
Hispanic	0.270	**<0.001**	**0.017**	0.640
Other	**<0.001**	**<0.001**	0.626	0.102
Missing	0.649	**<0.001**	0.499	**<0.001**

Bold *p*-values indicate significance at a 95% confidence level.

**Table A2:** A *p*-value table for respiratory system diagnoses-related opioid prescribing rate difference in each racial/ethnic group by time period.

Time Period	Compared to White				Compared to Black			Compared to Hispanic	
	Black	Hispanic	Other	Missing *	Hispanic	Other	Missing	Other	Missing
First period: 2012–2014	**<0.001**	**<0.001**	**<0.001**	0.161	0.156	0.058	0.263	0.779	0.356
Second period: 2012–2014	**<0.001**	0.300	**0.012**	**0.003**	**<0.001**	0.455	0.124	**0.005**	**0.001**
Third period: 2015–2017	0.059	0.198	**0.015**	**<0.001**	0.778	0.288	**<0.001**	0.636	**<0.001**

Bold *p*-values indicate significance at a 95% confidence level. * Proportional test outcomes for “Missing” racial/ethnic group are inconsistent due to the small sample sizes.

**Table A3:** A *p*-value table for injury-related opioid prescribing rate difference in each race/ethnic group by time period.

Time Period	Compared to White				Compared to Black			Compared to Hispanic	
	Black	Hispanic	Other	Missing *	Hispanic	Other	Missing	Other	Missing
First period: 2012–2014	**<0.001**	**0.012**	**<0.001**	0.999	**<0.001**	**<0.001**	0.689	**<0.001**	0.653
Second period: 2012–2014	**0.045**	0.246	**<0.001**	0.430	**0.021**	**<0.001**	0.909	**<0.001**	0.191
Third period: 2015–2017	**0.001**	**0.008**	**<0.001**	**<0.001**	**<0.001**	0.246	**0.003**	**<0.001**	**<0.001**

Bold *p*-values indicate significance at a 95% confidence level. * Proportional test outcomes for “Missing” racial/ethnic group are inconsistent due to the small sample sizes.
